# Supplementary material for: Topical MTH1 Inhibition Suppresses SKP2-WNT5a-Driven Psoriatic Hyperproliferation
Source: Int J Mol Sci. 2025 Jul 25;26(15):7174. doi: 10.3390/ijms26157174 (PMC12346197; doi:10.3390/ijms26157174)
Supplement: Supplementary file 1 [file ijms-26-07174-s001.zip › Supplemental figure legends.pdf]

SUPPLEMENTAL FIGURE LEGENDS

Suppl figure 1. No significant changes in the cell composition of the spleen after topical application of MTH1 inhibitor. Aldara cream (IMQ) was applied to the shaved backs of mice (C57B6/J) for 4 consecutive days. 4 h after IMQ treatment, the mice received daily topical application of the MTH1 inhibitor (TH1579) for 3 days (days 2-4). Flow cytometry analyses of (**a**) $CD3^+$ and (**b**) $Ly6G^+$ cells from spleens of TH1579- and IMQ-treated mice. mean ±SEM. n = 6.

Suppl figure 2: Flow cytometry analyses. Gating strategies for immunophenotyping of blood cells. Side scatter area (SSC-A) vs live/dead stained cells shows gate A encircling the live cells. The live cells are gated to diminish the doublets and coincidence events, gate B and the cells in the single gate are then gated into $CD19^+$/$CD19^-$ populations, and the $CD19^-$ events are further gated into $CD3^+$, NK^+, and NKt^+ cells. The $CD3^+$ cells are further divided into the different lymphocyte subsets $CD4^+$, $CD8^+$, and TCR γδ cells.
