# Supplementary material for: Topical MTH1 Inhibition Suppresses SKP2-WNT5a-Driven Psoriatic Hyperproliferation
Source: Int J Mol Sci. 2025 Jul 25;26(15):7174. doi: 10.3390/ijms26157174 (PMC12346197; doi:10.3390/ijms26157174)
Supplement: Supplementary file 1 [file ijms-26-07174-s001.zip › Supplemental methods.pdf]

SUPPLEMENTAL METHODS

Mass spectrometry sample preparation

Harvested TH1579-treated KCs were lysed and sonicated in RIPA buffer containing 1x protease inhibitor cocktail (ThermoFisher Scientific). Cell debris was removed by centrifuging at 13,000 rpm for 10 min at 4°C. Protein concentrations were determined using BCA assay (ThermoFisher Scientific). For each sample, 30 µg of total protein was used to prepare mass spec samples using Ultrasonic-Based Filter Aided Sample Preparation (US-FASP) [33]. Briefly, 10000 Da molecular weight cutoff (MWCO) filters were conditioned first with Milli-Q water and then with urea buffer (8M Urea in 0.1M Tris-HCl pH 8.5). The cell lysates were reduced with 10 mM Dithiothreitol (DTT) in a heat block at 95°C for 5 min. The reduced samples were added to the filters after mixing with up to 200 µl of urea buffer and centrifuged at 12000 g for 20 min. Proteins were alkylated by adding 100µl of 0.05 M Iodoacetamide in urea buffer to the filter units and sonicated for 5.25 min (7 cycles: 30 sec on and 15 sec off UT, 25% UA) to speed up the alkylation and then centrifuged at 12000 g for 20 min. Likewise, the digestion was performed with 100 µl of Pierce™ Trypsin/Lys-C Protease Mix (ThermoFisher Scientific) in 0.05M Tris-HCl pH 8.5 at an enzyme-to-protein ratio of 1:30. Peptides were desalted using Pierce C18 spin columns (ThermoFisher Scientific) and stored at -20°C after drying.

Liquid chromatography and mass spectrometry

The desalted and dried peptide samples were reconstituted in 0.1% Formic acid (FA) in ultra-pure Milli-Q water and the concentration was measured using a Nanodrop (Thermo Scientific). The samples were analysed using a nanoElute 2 LC system connected with a timsTOF HT mass spectrometer via the CaptiveSpray 2 source (Bruker, Bremen, Germany). The peptides were

separated by a 25 cm C18 column (150 µm inner diameter, 1.5 µm particle size, PepSep, Bruker Daltonics) with a gradient of 2-17 % solvent B (0.1% FA in acetonitrile (ACN)) in 25 min, 17-25% B in 5 min, 25-37% B in 5 min, 37-95% B in 10 min at flow rate of 400 nl/min. The MS data were acquired using the dia-PASEF method. The capillary voltage was set to 1600 V. The MS and MS/MS spectra were acquired from 100-1700 m/z. The ion mobility was scanned from 0.85 to 1.3 Vs/cm2. The ramp time was set to 100 ms. The collision energy was ramped linearly as a function of the mobility from 59 eV at 1/K0= 1.6 Vs/cm2 to 20 eV at 1/K0= 0.6 Vs/cm^2.

Protein identification and quantification

The raw DIA data files were converted into HTRMS files using the HTRMS Converter (version 18.7, Biognosys). These HTRMS files were processed using directDIA+(Deep) workflow in Spectronaut proteomics software (version 19.1.240724.62635, Biognosys). The data files were searched against the UniProtKB-proteome UP000005640 (Taxonomy: Homo sapiens, release: 2024-04-02, Entries: 82493). The default BGS Factory Settings were used for search and analysis with the following modifications: Trypsin/P and LysC/P were used as specific enzymes allowing for a maximum of two missed cleavages; Peptide length from 5 to 52; Carbamidomethylation of cysteines was set as fixed modification whereas Oxidation of methionine and N-acetylation were set as variable modifications; The identifications were filtered at FDRs of 0.01% on PSM, peptide and protein level; Excluded single-hit proteins; Missing values were imputed using global imputation strategy and cross-run normalisation was enabled.

Statistically differentially expressed proteins (DEPs) were identified using an unpaired student *t*-test with the cutoff of fold-change (FC) >1.5 (log2FC = 0.585) and *Q-value* (BH-FDR-corrected p-value) < 0.05.

Mass spectrometry - Data analysis

A nuanced approach was used to filter duplicate entries of proteins (distinct on account of different UniProt IDs) in the result of the unpaired Student t-test. Proteins were classified into five groups; proteins with adjusted p-value (Q-value) > 0.05 were assigned "non-significant" status. Proteins with a Q-value <0.05 and log2FC < -0.585 were assigned as "Downregulated (DE)" whereas those with Q-value <0.05 and log2FC> 0.585 were classified as "Upregulated (DE)". Similarly, proteins with a Q-value <0.05 and log2FC <0 were labelled "Downregulated" whereas those with log2FC> 0 were classified as "Upregulated". To retain only one instance of a protein, a priority-based scheme was employed. Criterion one: proteins with significant fold-change and adjusted p-values, were preferred over non-significant ones. Criterion two: peer-reviewed UniProt entries were given priority over non-peer-reviewed entries. Criterion three: complete records were given precedence against fragmented proteins. Lastly, in case there was a tie even after exhausting all the aforementioned criterions, the first entry was retained. Enrichment analysis was performed on the clean data using pathway databases KEGG, and Reactome. Additionally, we carried out an enrichment analysis on gene ontology (GO), which comprises of three distinct and orthogonal subontologies. We focused primarily on the "biological process" (BP). We used the R package clusterProfiler (4.12.6) for KEGG and GO enrichment analysis whereas ReactomePA (1.48.0) was used for Reactome pathway analysis.

Benjamin-Hochberg method was used for p-value adjustment with p-value cut-off set at 0.05.

All visualisations of enrichment results were implemented using the enrichplot (1.24.4) package. Volcano plot of DEPs was generated using ggplot2 (3.5.1), chord diagrams generated using the GOplot (1.0.2) package, and heatmap plot with unsupervised clustering was generated using ComplexHeatmap (2.20.0).

All downstream analysis and visualisation were performed in R using custom-written scripts.

qPCR analysis

The following predesigned TaqMan Gene expression assays were used: Il-*17a* (Mm00439618_m1), *Tnf* (Mm00443258_m1) and *Rplp0* (Mm00725448_s1) for mouse expression, and *SKP2* (Hs01021864_m1, *CDK4* (Hs00364847_m1), *CDKN1A* (Hs00355782_m1), *PCNA* (Hs00427214_g1)*,* *WNT5a* (Hs00998537_m1) and *RPLP0* (Hs99999902_m1) for human expression (ThermoFisher Scientific, Waltham, MA, USA).

Immunofluorescence

The following primary antibodies were used: CD45 (ab10588, Abcam, Cambridge, UK), Ly6B.2 (NBP2-13077, Novus Biologicals, Littleton, CO), PCNA (ab29, Abcam) and CD3 (ab5690, Abcam).

Cell viability assay

Cell viability was analysed after 72 h of TH1579-treatment (0.01-5 µM), using the PrestoBlue Cell viability reagent (Invitrogen, ThermoFisher Scientific), following the manufacturer's instructions. The OD readings were measured at 570/600 nm in a VersaMax microplate reader (Molecular Devices, Sunnyvale, CA). All samples were run in triplicate.

Crystal violet proliferation assay

The KCs were fixed in 4% formaldehyde for 10 min at room temperature. The cells were stained with 0.05% crystal violet for 20 min, washed with water, and air-dried. Then, dissolved in 1% sodium dodecyl sulfate (SDS) solution on a shaker for 3 h. The absorbance was measured at 550 nm, using a VersaMax microplate reader.

Flow cytometry on mouse spleen and skin-infiltrating immune cells

The fresh skin tissue was cut into small pieces and digested in RPMI 1640 medium (Gibco) containing 250 µg/ml Liberase TL (Roche Diagnostics, Mannheim, Germany) and 10 µg/ml DNase I (Sigma-Aldrich, St. Louis, MO) in for 2h at 37°C, 5% CO2. The solution was vortexed every 30 min. Digested skin was then mashed through a 70 µm mesh strainer, washed, and immediately stained for flow cytometry.

The dissected spleens were passed through a 70 µm mesh strainer, washed, and frozen in RPMI 1640 (Gibco, ThermoFisher Scientific) containing 20% FBS (Gibco, ThermoFisher Scientific) and 10% DMSO (Sigma-Aldrich). The cryopreserved cells were thawed and washed in 37ºC RPMI supplemented with 10%FBS and 25ku bensonase (Sigma-Aldrich). If contaminated with red blood cells, the cell pellet was processed with RBC Lysis buffer according to the manufacturer's instructions (Nordic Biosite, Stockholm, Sweden).

The skin and spleen cells were washed with PBS and live dead staining was performed according to the manufacturer's instructions. The samples were blocked with Mouse BD Fc block (Becton Dickinson, Stockholm, Sweden) for 10 min at room temperature and stained with antibodies mixed in 50µl Brilliant Stain Buffer (Becton Dickinson) for 30 min in the dark on ice. Following washes with PBS/0,2% BSA, the samples were analysed using the spectral flow cytometer Aurora (Cytek, San Diego, CA). 3-500 000 viable cells in the FS/SS-live/dead gate were collected from the digested dermis samples and 100 000 viable cells were collected from spleen cell samples for further analysis by Kalzua v2.1 (Beckman Coulter, Stockholm, Sweden).

Antibodies for flow cytometry analysis

Antibodies used for staining spleen cells were against mouse CD4-APC H7, F4/80-APC R700, NK1.1-PECy7, and Brilliant Stain Buffer were purchased from Becton Dickinson (Stockholm,

Sweden). Antibodies against mouse TCRγ/δ -BrilliantViolett 421, CD19-Spark Blue550, CD3-FITC and CD8-BV570 was purchased from Nordic Biosite. Antibodies used for staining immune infiltrating cells in the dermis were anti-mouse TCRγ/δ-BrilliantViolett 421 purchased from Nordic Biosite and anti-mouse CD45-AlexaFlour700 and anti-mouse CD3-PerCP-Cy5.5 from Becton Dickinson.

20% normal rat serum (Jackson ImmunoResearch Laboratories, Ely, UK) or FC mouse block (Becton Dickinson) was added to the cell suspension prior to immunostaining. All antibodies were titrated and fluorescence-minus-one (FMO) controls performed [34]. Live/dead estimation was based on Fixable Viability Stain 510 BD Horizon™ (Becton Dickinson).

Gating Strategy – Flow cytometry

All events not stained by Fixable Viability Stain 510 were plotted on 2 morphological plots to exclude aggregates and coincidental events. The cells taken from the digested skin samples were further plotted $CD45^+$, $CD3^+$ and $CD\gamma\delta^+$. The cells from spleen samples were separated sequentially as $CD19^+$ $CD19^-$, $CD19^-$ further analysed as $CD3^+$, $NK1.1^+$, $CD4^+$ $CD8^+$, $CD3^+CD4^-$, and $CD8^{--}$ and finally TCR-gd^+, and $F4/80^+$ cells (Suppl fig. 2).
