# Supplementary figures and images for: Topical MTH1 Inhibition Suppresses SKP2-WNT5a-Driven Psoriatic Hyperproliferation
Source: Int J Mol Sci. 2025 Jul 25;26(15):7174. doi: 10.3390/ijms26157174 (PMC12346197; doi:10.3390/ijms26157174)

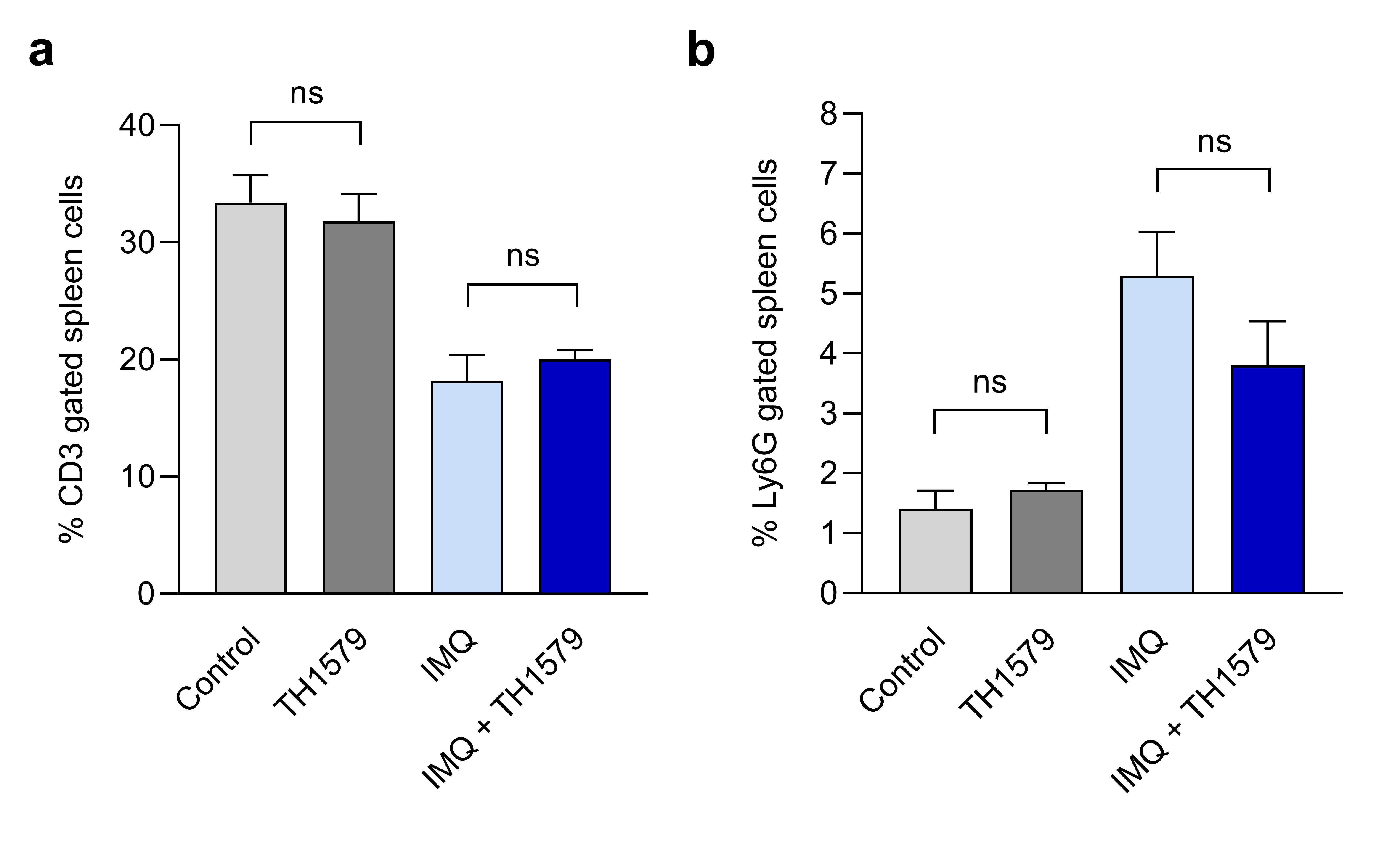

Supplement: Supplementary file 1 [file ijms-26-07174-s001.zip › Suppl figure S1.jpg]

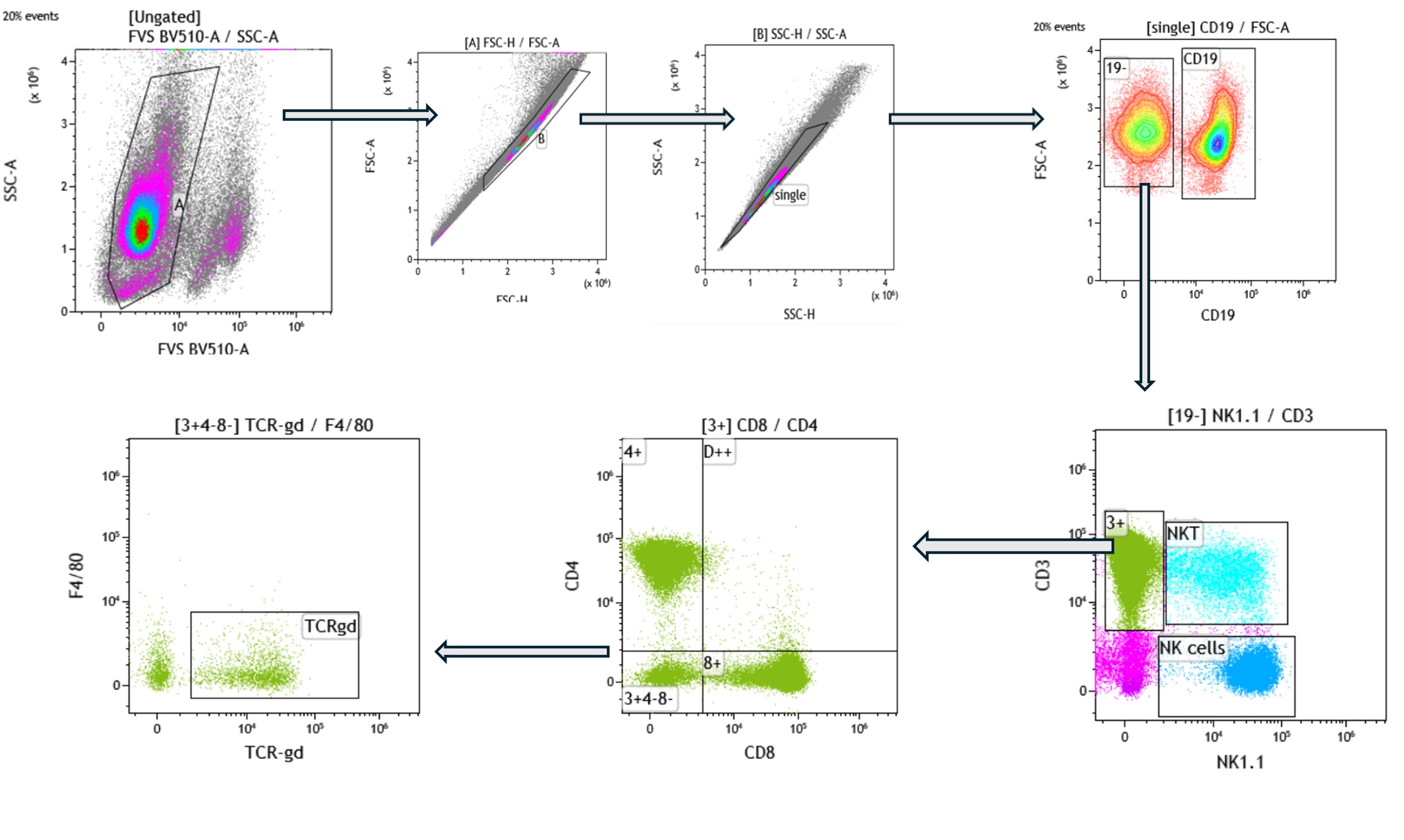

Supplement: Supplementary file 1 [file ijms-26-07174-s001.zip › Suppl figure S2.jpg]
